# Angiotensin II Receptor Blocker Ameliorates Stress-Induced Adipose Tissue Inflammation and Insulin Resistance

**DOI:** 10.1371/journal.pone.0116163

**Published:** 2014-12-31

**Authors:** Motoharu Hayashi, Kyosuke Takeshita, Yasuhiro Uchida, Koji Yamamoto, Ryosuke Kikuchi, Takayuki Nakayama, Emiko Nomura, Xian Wu Cheng, Tadashi Matsushita, Shigeo Nakamura, Toyoaki Murohara

**Affiliations:** 1 Department of Cardiology, Nagoya University Graduate School of Medicine, Nagoya, Japan; 2 Department of Clinical Laboratory, Nagoya University Hospital, Nagoya, Japan; 3 Department of Blood Transfusion, Nagoya University Hospital, Nagoya, Japan; 4 Department of Pathology, Nagoya University Hospital, Nagoya, Japan; 5 Department of Blood Transfusion, Aichi Medical University Hospital, Nagakute, Japan; Kobe University, Japan

## Abstract

A strong causal link exists between psychological stress and insulin resistance as well with hypertension. Meanwhile, stress-related responses play critical roles in glucose metabolism in hypertensive patients. As clinical trials suggest that angiotensin-receptor blocker delays the onset of diabetes in hypertensive patients, we investigated the effects of irbesartan on stress-induced adipose tissue inflammation and insulin resistance. C57BL/6J mice were subjected to 2-week intermittent restraint stress and orally treated with vehicle, 3 and 10 mg/kg/day irbesartan. The plasma concentrations of lipid and proinflammatory cytokines [Monocyte Chemoattractant Protein-1 (MCP-1), tumor necrosis factor-α, and interleukin-6] were assessed with enzyme-linked immunosorbent assay. Monocyte/macrophage accumulation in inguinal white adipose tissue (WAT) was observed with CD11b-positive cell counts and mRNA expressions of CD68 and F4/80 using immunohistochemistry and RT-PCR methods respectively. The mRNA levels of angiotensinogen, proinflammatory cytokines shown above, and adiponectin in WAT were also assessed with RT-PCR method. Glucose metabolism was assessed by glucose tolerance tests (GTTs) and insulin tolerance tests, and mRNA expression of insulin receptor substrate-1 (IRS-1) and glucose transporter 4 (GLUT4) in WAT. Restraint stress increased monocyte accumulation, plasma free fatty acids, expression of angiotensinogen and proinflammatory cytokines including MCP-1, and reduced adiponectin. Irbesartan reduced stress-induced monocyte accumulation in WAT in a dose dependent manner. Irbesartan treatment also suppressed induction of adipose angiotensinogen and proinflammatory cytokines in WAT and blood, and reversed changes in adiponectin expression. Notably, irbesartan suppressed stress-induced reduction in adipose tissue weight and free fatty acid release, and improved insulin tolerance with restoration of IRS-1 and GLUT4 mRNA expressions in WAT. The results indicate that irbesartan improves stress-induced adipose tissue inflammation and insulin resistance. Our results suggests that irbesartan treatment exerts additive benefits for glucose metabolism in hypertensive patients with mental stress.

## Introduction

Modern stressors are closely related to psychological threat (e.g., work stress, social anxiety, and natural disasters) in daily life and often sustained. Epidemiological studies have demonstrated that chronic mental stress in modern lifestyle is closely linked to the incidence of hypertension, cardiovascular disease, metabolic syndrome (MetS), and diabetes mellitus [1]. Especially, there is substantial overlap between diabetes and hypertension in etiology. Accumulating evidence has demonstrated associations of disturbed psychophysiological responses with sub-clinical measures of atherosclerosis, hypertension, and metabolic risk [2]. As the onset of diabetes is closely linked to cardiovascular complications in hypertensive patients, stress-related disorders would be a potential therapeutic target in hypertensive patients. Stress activates the sympathetic nervous system (SNS) and the hypothalamic-pituitary-adrenal (HPA) axis to alternate systemic hormonal and immune responses [3], resulting in negative health effects on glucose metabolism, leading to the onset of type 2 diabetes [4].

We investigated recently how stressors perturb homeostasis of glucose metabolism, and found that the pathophysiological mechanism involved in this process is quite similar to that in the obesity-related MetS [5]. Using a murine model, we demonstrated that two-week intermittent restraint stress enhanced chronic inflammation of the adipose tissue and resulted in impairment of insulin sensitivity [5]. Furthermore, chronic stress promoted the secretion of adrenal catecholamines and glucocorticoids, resulting in lipolysis in visceral adipose tissue with free fatty acid (FFA) release [5]. Chronic FFA release stimulated toll-like receptor 4 on adipocytes to produce inflammatory adipokines, including tumor necrosis factor-α (TNF-α), interleukin-6 (IL-6), and monocyte chemoattractant protein-1 (MCP-1), and exacerbate monocyte accumulation, giving rise to impaired insulin sensitivity. MCP-1 inhibition prevented visceral adipose inflammation and insulin resistance in this murine stress model [5] as well as a in MetS murine model [6].

The renin-angiotensin system (RAS) is classically known for its role in systemic regulation of blood pressure, fluid and electrolyte balance, and has been recognized as an established therapeutic target for hypertension. Angiotensin II receptor blockers (ARBs) are used as one of the first-choice drugs for patients with hypertension. Numerous clinical trials demonstrate that ARBs improve glucose metabolism and delay the onset of diabetes mellitus in hypertensive patients [7]. Therefore the current study have focused on how the RAS is involved in obese-induced insulin resistance [6]. Obesity, which is one of the main features of the MetS, is associated with overactivation of both systemic and adipose RAS in human and animals [8]. White adipose tissue (WAT) expresses traditional RAS and is a predominant source of angiotensinogen, which activates the local RAS in an autocrine manner in response to weight gain [8]. Activation of adipose RAS contributes to adipose tissue inflammation and inhibits insulin metabolic signaling [8]. The SNS as well as the RAS are also activated in obesity, and both systems can upregulate the actions of the other [9]. It is assumed that activation of the adipose RAS exacerbates stress-induced adipose inflammation in combination with SNS activation, and is another therapeutic target for stress-induced adipose inflammation.

In a manner similar to the obesity-induced MetS, it is also anticipated that manipulation of both the RAS and MCP-1 is a potential target for stress-induced insulin resistance [5] [10]. Angiotensin II induces MCP-1 via the AT1 receptor through activation of RhoA-dependent and redox-sensitive pathways to facilitate monocyte accumulation [11]. Reportedly the angiotensin II type 1 receptor blocker (ARB) irbesartan inhibits MCP-1 production, and acts as a potent antagonist of the MCP-1 receptor, CC motif chemokine receptor 2 (CCR2), due to its molecular structure, in addition to its original AT1 receptor blocking effect [9]. Based on these results, it is predicted that irbesartan strongly suppresses stress-induced adipose inflammation through synergetic combination of the RAS and MCP-1/CCR2 pathway. In the present study, we investigated the outcome of treatment with irbesartan in a murine stress model, with special emphasis on the suppression of stress-induced adipose inflammation and insulin resistance.

## Materials and Methods

For more complete description, see Materials S1.

### Animals

All animals, obtained from Chubu Kagaku Shizai Co., Ltd (Nagoya, Japan), were housed in the Division for Research of Laboratory Animals, Nagoya University Graduate School of Medicine. The animal protocols were approved by the Institutional Animal Care and Use Committee of Nagoya University (Protocol Number 26183), and performed according to the Guide for the Care and Use of Laboratory Animals published by the National Institutes of Health.

### Restraint stress procedure

Eight-week-old male C57BL/6J mice were randomly assigned to the control (n = 20) and the stress group (n = 30). Control mice were left undistributed, while stressed mice were individually subjected to 2 h/day of immobilization stress for two weeks, as described previously [5] [12] [13]. Within each group, mice were randomly assigned to receive either vehicle alone (0.4% methylcellulose), or two doses of oral irbesartan (3 or 10 mg/kg/day, generous gift from Sumitomo Dainippon Pharma Co.) for 2 weeks. The control animals were given vehicle or the higher dose of irbesartan (n = 10, respectively). The stressed animals were given vehicle, or lower or higher dose of irbesartan (n = 10, respectively). Body weight and food intake were monitored during this period. After the 2-week restraint, systolic blood pressure was measured. Before euthanasia, animals were anesthetized (intraperitoneal sodium pentobarbital, 150 mg/kg), and then biological samples were collected for total RNA extraction, analysis for plasma lipid composition [14] and pathology.

### Quantitative PCR

Total RNA extraction, reverse-transcription, and quantitative PCR were performed as described previously [15]. The primer sequences used in this study are listed in Supporting Material. The amount of each RNA was normalized to the respective β-actin mRNA.

### Histological analysis

The inguinal WAT was processed for hematoxylin-eosin (H&E) and CD11b staining using standard histological procedures [5]. Two investigators blindly and independently measured the size of inguinal adipocytes and counted the number of CD11b-positive and -negative cells under a microscope at×200 magnification. Ten microscopic fields were chosen in three different sections per mouse for examination.

### Enzyme-linked immunosorbent assay

Serum levels of MCP-1, TNF-α and IL-6 were quantified using Mouse CCL2 ELISA Ready-SET-Go (Human CCL2 for detection of 7ND; eBioscience, Kobe, Japan), mouse insulin (Mercodia, Uppsala, Sweden), TNF-α and IL-6 ELISA kit (R&D Systems, Minneapolis, MN), respectively, according to the instructions provided by the manufacturer.

### Intraperitoneal glucose and insulin tolerance tests

After two weeks of daily stress, mice were subjected to an intraperitoneal glucose tolerance test (GTT) and insulin tolerance test (ITT) using standard methods [5]. Briefly, for GTT, mice were fasted overnight and then challenged with 2 g/kg D-glucose (Sigma-Aldrich, St. Louis, MO), followed by serial assessment of blood glucose up to 120 min using a blood glucose level monitor (Glutest Ace, Sanwa Kagaku Kenkyusho Co, Nagoya, Japan). For ITT, the mice were fasted for 16 hours before testing. Insulin (0.75 U/kg, Actrapid Penfill, NovoNordisk, Copenhagen, Denmark) was injected intraperitoneally, and blood glucose was measured.

### Statistical analysis

Data are expressed as mean±SD. Differences between groups were assessed by one-way ANOVA followed by Fisher’s test, and considered significant at *P*<0.05. Frequencies were analyzed by the chi-squared test.

## Results

### Irbesartan prevents stress-induced adipose inflammation

**Figure 1:**
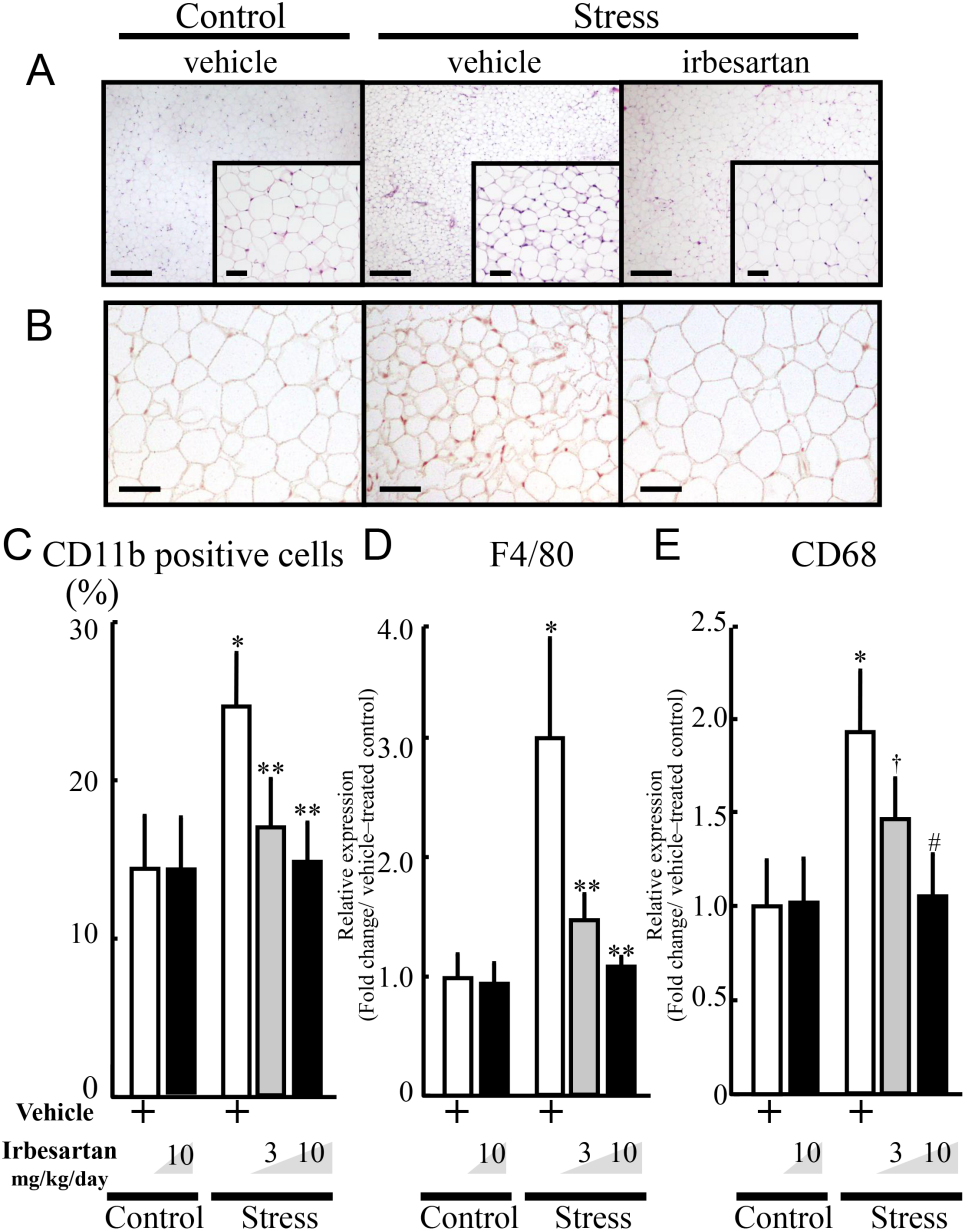
Accumulation of monocytes in inguinal adipose of stressed mice. Stressed mice were individually subjected to 2 h/day of immobilization stress for two weeks. Animals received oral vehicle, 3, or 10 mg/kg/day of irbesartan during the same period. Inguinal adipose tissues from stressed and control (non-stressed) mice were analyzed by H&E staining (A), CD11b immunostaining (B and C), and quantitative RT-PCR for CD68 and F4/80 (D and E). **A:** Accumulation of mononuclear cells in inguinal adipose tissues following the 2-week restraint stress. Top panel, ×40 magnification, bar = 250 µm. Inset, ×200 magnification, bar = 50 µm. **B:** Increased accumulation of CD11b-positive cells (monocytes) in adipose tissue of stressed mice (×200 magnification, bar = 50 µm). **C:** Quantitative analysis of CD11b-positive cells relative to total nuclear number. Data are mean±SD. n = 10 for all the groups.*P<0.001, compared with the vehicle-treated control mice, **P<0.001, compared with the vehicle-treated and stressed mice. **D** and **E**: Quantitative analysis of F4/80 (D) and CD68 (E) expression levels in adipose tissue. Data are mean±SD. n = 10 for all the groups. Values are expressed relative to the vehicle-treated control mice. (**D**) *P<0.001, compared with the vehicle-treated control mice, **P<0.001, compared with the vehicle-treated and stressed mice, respectively. (**E**) *P<0.001, compared with the vehicle-treated control mice, ^†^ *P<*0.012, compared with vehicle-treated and stressed mice, ^#^ *P<*0.02, compared with the stressed mice treated with a lower dose of irbesartan (3 mg/kg/day), respectively.

Examination of C57BL/6J mice subjected to 2 weeks of daily restraint stress showed significant increase in mononuclear cell infiltration, CD11b-positive cells and the mRNA expression levels of monocyte/macrophage cell surface markers (F4/80 and CD68) in inguinal adipose tissues (Fig. 1). Further examination of the effects of oral irbesartan at 3 and 10 mg/kg/day showed no change in blood pressure, in agreement with previous reports (data not shown) [16]. However, irbesartan significantly reduced monocyte accumulation and the mRNA expression levels of monocyte surface markers in adipose tissues of stressed mice, and these effects were dose-dependent. Even when used at the higher dose, irbesartan neither altered monocyte accumulation nor the mRNA expression of surface markers in the control mice.

### Irbesartan reduces stress-induced angiotensinogen level

**Figure 2:**
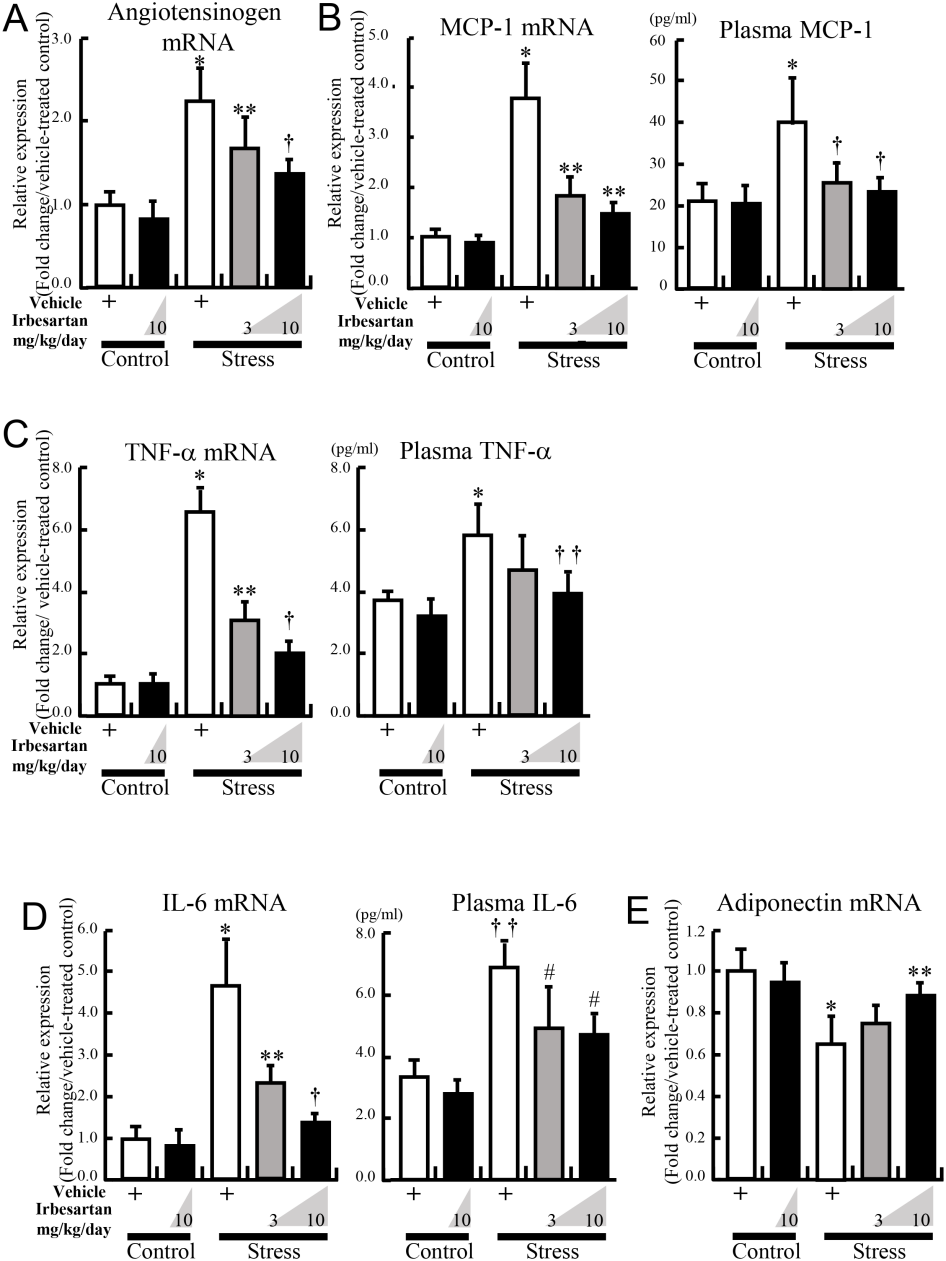
Irbesartan reduced the expression of stress-induced proinflammatory adipokines and restored adiponectin expression in adipose tissue. Inguinal adipose tissues from control mice treated with vehicle or irbesartan (10 mg/kg/day), and stressed mice treated with vehicle or irbesartan (3 or 10 mg/kg/day) were analyzed by quantitative RT-PCR for angiotensinogen (**A**), MCP-1 (**B**), TNF-α (**C**), IL-6 (**D**), and adiponectin (**E**). Values are expressed relative to the vehicle-treated control mice. Plasma levels of MCP-1, TNF-α, and IL-6 from these groups were also measured. Data are mean ± SD of 10 mice for RT-PCR, 6 mice for ELISA per group. (**A**) *P<0.001, compared with the vehicle-treated control mice, **P<0.046, compared with the vehicle-treated and stressed mice, ^†^ *P<*0.042, compared with the stressed mice treated with a lower dose of irbesartan (3 mg/kg/day), respectively. (**B**) *P<0.001, compared with the vehicle-treated control mice, **P<0.001, compared with the vehicle-treated and stressed mice, ^†^ *P<*0.003, compared with the vehicle-treated and stressed mice, respectively. (**C**) *P<0.001, compared with the vehicle-treated control mice, **P<0.001, compared with the vehicle-treated and stressed mice, ^†^ *P<*0.004, compared with the stressed mice treated with a lower dose of irbesartan (3 mg/kg/day), ^††^ *P<*0.05, compared with the vehicle-treated and stressed mice, respectively. (**D**) *P<0.001, compared with the vehicle-treated control mice, **P<0.003, compared with the vehicle-treated and stressed mice, ^†^ *P<*0.004, compared with stressed mice treated with a lower dose of irbesartan (3 mg/kg/day), ^††^ *P<*0.002, compared with the vehicle-treated control mice, ^#^ *P<*0.02, compared with the vehicle-treated and stressed mice, respectively. (**E**) *P<0.001, compared with the vehicle-treated control mice, **P<0.05, compared with the vehicle-treated and stressed mice, respectively.

In the vehicle-treated stressed mice, the mRNA expression level of angiotensinogen in adipose tissues was more than double that of the vehicle-treated control mice (Fig. 2A). Irbesartan reduced angiotensinogen production in a dose dependent manner (Fig. 2A).

### Irbesartan reduces inflammatory adipokine levels in stressed mice

The 2-week restraint stress resulted in significant increases in the mRNA expression levels of MCP-1, TNF-α, and IL-6 in adipose tissues, and these changes were suppressed in a dose dependent manner of irbesartan (Fig. 2B–D). Irbesartan also decreased the elevated levels of plasma MCP-1, TNF-α, and IL-6 in the stressed mice, in parallel with the changes in their mRNA expression levels in adipose tissue. The stress-induced decrease in the mRNA expression level of adiponectin was inversely increased by the treatment (Fig. 2E). However, no changes in the expression levels of these adipokines were noted in adipose tissue of control mice treated with vehicle or higher-dose irbesartan.

### Irbesartan reduces stress-induced lipolysis

**Figure 3:**
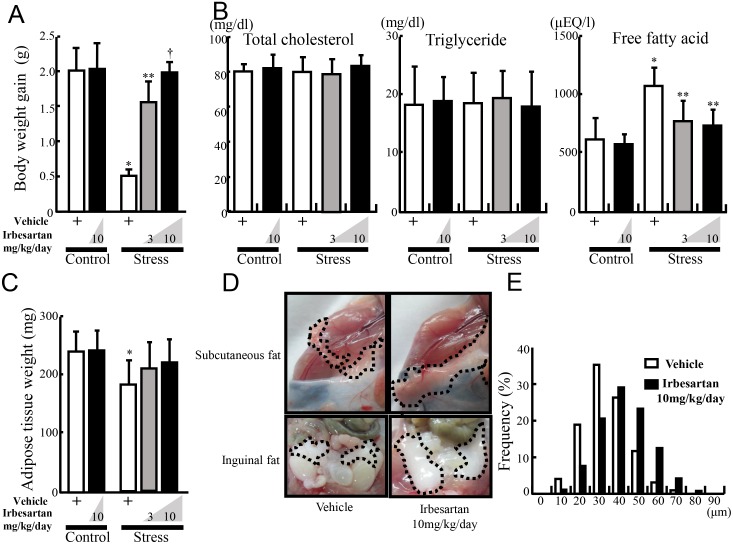
Irbesartan restored stress-induced decrease in weight gain and reduced adipose tissue weight. Body weight and inguinal adipose tissue of the control and stressed mice were weighed before and after the stress period, and the cell size in the collected adipose tissue was estimated under a microscope at×200 magnification using image analysis software. **A:** Body weight gain in the control mice with or without irbesartan treatment (10 mg/kg/day) and stressed mice with or without irbesartan treatment (3 or 10 mg/kg/day). *P<0.001, compared with the vehicle-treated control mice, **P<0.01, compared with the vehicle-treated and stressed mice, ^†^ *P<*0.001, compared with the vehicle-treated and stressed mice. **B:** Plasma fat and fatty acid composition in the control mice with or without irbesartan treatment (10 mg/kg/day) and stressed mice with or without irbesartan treatment (3 or 10 mg/kg/day). *P<0.01, compared with the vehicle-treated control mice, **P<0.05, compared with the vehicle-treated and stressed mice. **C:** Inguinal adipose tissue weight in the control mice with or without irbesartan treatment (10 mg/kg/day) and stressed mice with or without irbesartan treatment (3 or 10 mg/kg/day). * *P*<0.03, compared with the vehicle-treated control mice. **D:** Subcutaneous and inguinal fat pad. Circle dot line: adipose tissue. **E:** Distribution of adipocyte size in inguinal adipose tissues of stressed mice with or without irbesartan (10 mg/kg/day) treatment. Data are mean ± SD of 10 mice per group.

Two-week higher-dose irbesartan did not alter body weight gain of control mice (Fig. 3A). On the other hand, body weight gain was significantly reduced in stressed animals after the 2-week-stress period, and irbesartan restored the stress-induced decrease in body weight gain in a dose-dependent manner. However, mice of each group consumed almost similar amount of food (approximately 127 mg/g/day).

Analysis of plasma lipid profile showed that stress and irbesartan treatment did not alter total cholesterol or triglyceride levels (Fig. 3B). On the other hand, FFA concentration was increased in stressed mice, and irbesartan treatment significantly reduced these concentrations in a dose dependent manner (Fig. 3B). The weight of inguinal adipose tissue was significantly less in the stressed mice than the control, and this decrease was recovered by irbesartan (Fig. 3C). Indeed, stress-induced reduction in subcutaneous and inguinal fat and the decrease in adipocyte size were restored by irbesartan (Fig. 3D and E). The above results indicate that irbesartan reduces stress-induced lipolysis and FFA release.

### Irbesartan rescues stress-induced insulin insensitivity

**Figure 4:**
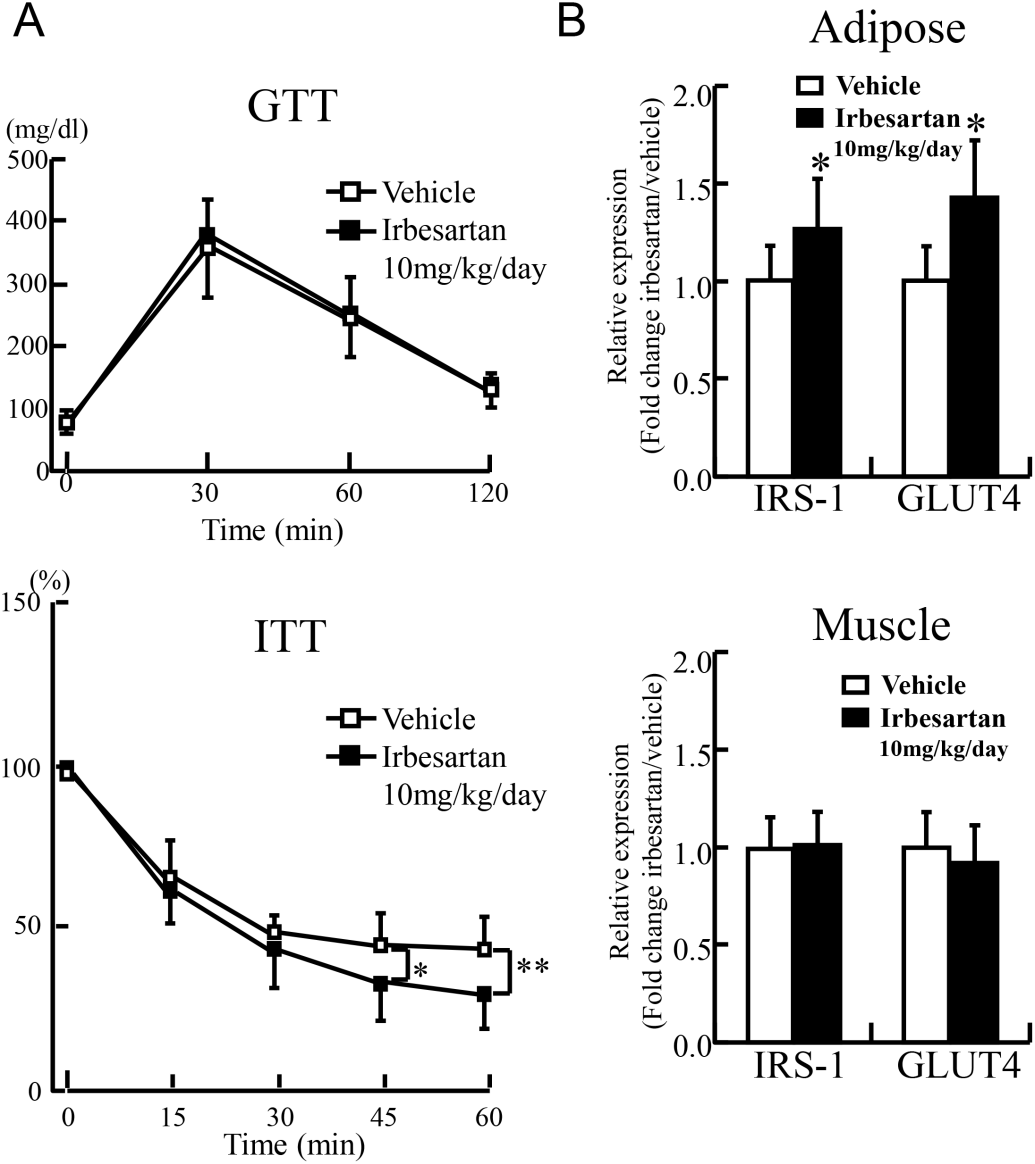
Irbesartan rescued stress-induced decline in insulin sensitivity. **A:** Glucose tolerance was comparable between the stressed mice treated with vehicle and irbesartan (10 mg/kg/day) after stress. Insulin tolerance showed significant recovery in the irbesartan-treated and stressed mice (lower panel). Data are mean ± SD of 10 mice per group. *P<0.05, and **P<0.02, compared with the vehicle-treated and stressed mice. **B:** Quantitative analysis of IRS-1 and GLUT4 expression in inguinal adipose tissue and skeletal muscle (adductor muscle) of the stressed mice treated with vehicle or irbesartan (10 mg/kg/day). Data are mean ± SD of 10 mice per group. *P<0.05, compared with the vehicle-treated and stressed mice.

We reported previously that stress reduces insulin sensitivity, and this effect was restored by MCP-1 inhibition [5]. To test whether irbesartan treatment could also improve exacerbation of glucose metabolism, we measured GTT, ITT, and insulin receptor substrate-1 (IRS-1) and glucose transporter-4 (GLUT-4) mRNA expression levels in adipose tissues and skeletal muscles (adductor muscles). There was no significant difference in glucose tolerance between vehicle and irbesartan after stress (Fig. 4A). However, insulin tolerance improved significantly after 45 min in the higher-dose irbesartan group (Fig. 4A). We could not find significant changes in GTT and ITT in the lower-dose irbesartan group although insignificant improvements were observed after 60 min in ITT (data not shown). We also observed that a higher dose of irbesartan restored the mRNA expression levels of IRS-1 and GLUT-4 in inguinal adipose tissues (Fig. 4B). The mRNA expression levels of IRS-1 and GLUT-4 in skeletal muscle were not altered by the treatment. Considered together, the above findings indicate that irbesartan suppresses stress-induced lipolysis and adipose inflammation to improve glucose metabolism.

## Discussion

The main finding of this study is that irbesartan suppressed stress-induced adipose inflammation to restore insulin sensitivity. In the present study, two-week intermittent restraint stress resulted in low-grade inflammation, decreased body weight gain [5] and increased angiotensinogen mRNA expression level in murine adipose tissue. Irbesartan administered at 3 or 10 mg/kg/day markedly suppressed stress-induced adipose inflammation and induction of angiotensinogen. Notably, irbesartan markedly suppressed stress-induced lipolysis and prevented changes in adipocyte size without changing food intake. Irbesartan also improved insulin sensitivity with restoration of adipose IRS-1 and GLUT-4.

The RAS and the SNS mediate responses to psychological stress. Chronic psychological stressors are reported to increase circulating plasma renin and angiotensin II, resulting in sustained systemic pro-inflammatory tendency [4]. In agreement with this finding, we also demonstrated that chronic stress induced adipose angiotensinogen to activate systemic and adipose RAS. Angiotensinogen is secreted in many organs including liver, kidney, and vascular cells [17]. Since adipose-derived angiotensinogen in rodents contributes to one third of the circulating angiotensinogen [17] and diet-induced obesity does not alter angiotensinogen expression in the aorta, kidney, and liver [18], the increase in adipose-derived angiotensinogen is more likely to be responsible for both systemic and adipose RAS activation in stressed subjects. Adipose-derived angiotensinogen is positively regulated by angiotensin II [17], sympathetic nerve activation [9], insulin [19], and inflammatory adipokines, including TNF-α [20], and IL-6 [21]. In stressed subjects, SNS activation stimulates adipose tissue to induce angiotensinogen, resulting in initiation of adipose RAS activation [9]. Adipose RAS activation induces inflammatory adipokines and angiotensinogen for autocrine activation. AT_1_ receptor stimulation is a potent inducer of MCP-1 [17], which plays a critical role in stress-induced adipose inflammation [5]. Furthermore, stress-induced insulin resistance could also contribute to the increase in angiotensinogen [11]. Thus, the RAS and inflammatory pathway synergistically exacerbate adipose inflammation through a positive feedback. Irbesartan is a potent ARB, which possesses higher affinity for CCR2 based on molecular modeling, and inhibits MCP-1 production via NFκB activation [16]. Treatment with irbesartan diminished adipose angiotensinogen expression and inhibited the MCP-1/CCR2 pathway, resulting in breaking the vicious circle of stress-induced adipose RAS activation [22].

In stressed subjects, stress-induced cortisol release and adipose SNS activation initiate lipolysis, resulting in reduction in adipose cell size and increase in FFA concentration [5], [23], [24]. Stress-induced lipolysis and FFA release can initiate stress-induced adipose inflammation[5]. Furthermore, chronic inflammation of the adipose tissue also accelerates unregulated lipolysis (Fig. 2B). Macrophage-derived TNF-α, which is derived from infiltrated macrophages and adipocytes in WAT, acts on TNF-α receptor in hypertrophied adipocytes, thereby inducing proinflammatory cytokine production and adipocyte lipolysis via NF-κB and MAPK-dependent mechanisms, respectively [25]. In the present study, irbesartan reduced monocyte accumulation and TNF-α induction, and thus broke the vicious circle between stress-induced lipolysis and adipose tissue inflammation (Fig. 3).

Irbesartan improved stress-induced insulin resistance via anti-inflammatory and pleiotropic signaling effects. We previously reported that restraint stress induces lipolysis and FFA release to induce low-grade WAT inflammation [5]. This pathological mechanism is quite similar to that of metabolic syndrome. We furthermore demonstrated that MCP-1 inhibition with 7ND and its antibody suppressed stress-induced adipose inflammation, resulting in significant improvements in insulin sensitivity [5]. In the present study, we demonstrated that the irbesartan treatment suppress stress-induced MCP-1 induction and adipose inflammation, and improved insulin resistance.

Since RAS and insulin signaling share the PI3 kinase pathway and tyrosine phosphorylation of IRS-1 after binding to their respective receptors [11], treatment with irbesartan would systemically improve insulin sensitivity in a post-transcriptional manner. Moreover, irbesartan is also known as a selective modulator of peroxisome proliferator-activated receptor (PPAR)-γ [26], and treatment with irbesartan in the present study improved both insulin sensitivity and adipose inflammation in stressed animals. Irbesartan is also known to restore glucose metabolism in accordance with expression levels of IRS-1 and GLUT4 in adipose tissues through significant suppression of adipose TNF-α [27] [28]. This would also alter systemic insulin sensitivity because adipose GLUT4 is closely linked to insulin sensitivity in skeletal muscles and liver [29]. Irbesartan suppressed adipose-derived FFA release, which upregulates the Toll-like receptor network in skeletal muscles, resulting in improvement of insulin sensitivity [30]. Any decrease in plasma IL-6 would be also anticipated to functionally improve insulin signaling in skeletal muscles at IRS-1function, which is independent from IRS-1 and GLUT4 expression [31]. Restored adiponectin level in adipose tissue should also improve systemic insulin sensitivity [32].

Evidence from a population cohort study suggests that psychological stress is a cardiovascular risk [2]. Accumulating evidence has demonstrated the association of disturbed psychophysiological responses with cardiovascular risk factors, including sub-clinical measures of atherosclerosis, such as endothelial dysfunction, hypertension, and impaired glucose and lipid metabolism [2]. Today’s patients with hypertension, who are under persistent stress, often develop insulin resistance [1].Treatment with irbesartan should improve insulin sensitivity in at least a subgroup of hypertensive patients with mental stress and be linked to better clinical outcome beyond blood pressure control [33].

In limitation, we could not clearly specify the direct causal mechanism how the irbesartan treatment improved insulin sensitivity in the stressed individuals because irbesartan affects pleiotropic pathways, including the RAS, inflammatory cytokines such as MCP-1/CCR2, and PPAR-γ. As shown above, it has been reported that irbesartan possesses multifactorial anti-inflammatory properties, and that reduced adipose inflammation is strongly linked to the restoration in insulin sensitivity. The recent study has shown that anti-inflammatory effects of ARB restore systemic insulin sensitivity in non-diabetic hypertensive patients [34]. The present study would shed light on the new benefit of the irbesartan treatment to the modern stressed and hypertensive patients.

In conclusion, we demonstrated that irbesartan inhibits stress-induced adipose inflammation via regulation of the RAS and inflammatory cytokines, and inhibition of lipolysis. The synergistic anti-inflammatory effects restored insulin sensitivity in the stressed animals.

## Supporting Information

S1 Materials (DOCX)
