# Supplementary material for: Angiotensin II Receptor Blocker Ameliorates Stress-Induced Adipose Tissue Inflammation and Insulin Resistance
Source: PLoS One. 2014 Dec 31;9(12):e116163. doi: 10.1371/journal.pone.0116163 (PMC4281136; doi:10.1371/journal.pone.0116163)
Supplement: S1 Materials — (DOCX) [file pone.0116163.s001.docx]

**ONLINE SUPPLEMENT**

**Angiotensin II Receptor Blocker Ameliorates Stress-induced Adipose Tissue Inflammation and Insulin Resistance**

Motoharu Hayashi^1^, Kyosuke Takeshita^12^, Yasuhiro Uchida^1^, Koji Yamamoto^3^, Ryosuke Kikuchi^2^, Takayuki Nakayama^5^, Emiko Nomura^3^ Xian Wu Cheng^1^, Tadashi Matsushita^3^, Hideo Nakamura^4^, and Toyoaki Murohara^1^

^1^Department of Cardiology; Nagoya University Graduate School of Medicine, Nagoya, Japan; Departments of ^2^Clinical Laboratory; ^3^Blood Transfusion; and ^4^Pathology, Nagoya University Hospital, Nagoya, Japan. ^5^Department of Blood Transfusion; Aichi Medical University Hospital, Nagakute, Japan.

*Running title*: ARB improves stress-induced insulin resistance

Correspondence to: A/Prof. Kyosuke Takeshita, MD, PhD, FAHA, Department of Cardiology Nagoya University Graduate School of Medicine, 65 Tsurumai-cho Nagoya, Aichi 466-8550, Japan.

Tel: +81 52 744 2147; Fax: +81 52 744 2138

E-mail: [kyousuke@med.nagoya-u.ac.jp](mailto:kyousuke@med.nagoya-u.ac.jp)

**SUPPLEMENTARY METHODS SECTION**

**Materials and Methods**

***Animals and restraint stress procedure***

Eight-week-old male C57BL/6J mice (Chubu Kagaku Shizai Co, Nagoya, Japan) were housed two per cage under standard conditions (23±1°C, 50±5% humidity), with a 12-h light/dark cycle in a viral pathogen-free facility and handled properly, as described in our previous study [[1](#_ENREF_1)] [[2](#_ENREF_2)]. Animals were randomly assigned to the control or stress group. Control mice were left undistributed and allowed contact with each other, while stressed mice were kept in individual cages and subjected to 2 h/day of immobilization stress over a period of two weeks (between 10 am and 12 noon, 6 days a week), as described previously [[3](#_ENREF_3)]. In brief, chronic stress was applied using a 50-ml conical centrifuge tube with multiple punctures designed for close fit of the individual mouse. Within each group, mice were randomly assigned to receive either vehicle alone (0.4% methylcellulose) or irbesartan (3.0 or 10 mg/kg/day, a generous gift from Sumitomo Dainippon Pharma Co.) orally, 10 mice per group, for 2 weeks.

Body weight and food intake were monitored during this period. After the 2-week restraint, mice were euthanized and blood, inguinal adipose tissue, and skeletal muscle (adductor muscles) samples were obtained for analysis. Plasma total cholesterol, triglyceride, and free fatty acid (FFA) levels were measured with commercially available enzymatic kit (LabAssay™ Cholesterol, LabAssay™ Triglyceride and LabAssay™ NEFA, respectively, Wako, Japan) [[4](#_ENREF_4)]. All studies were completed between 10 am and 12 noon, and conducted before and after the 2-week treatment. Systolic blood pressure was measured by the tail-cuff method (BP-98A, Softron, Tokyo, Japan) as described previously [[5](#_ENREF_5)].

**Quantitative PCR**

Total RNA extraction, reverse-transcription, and quantitative PCR were performed as described previously [[2](#_ENREF_2),[6](#_ENREF_6)]. The primer sequences used in this study are listed in Supplementary Table 1.

**Histological analysis of inguinal adipose tissue**

The inguinal white adipose tissue was processed and 5-μm thick sections were stained with H&E using standard histological procedures. Stained slides were examined microscopically by two independent investigators who were blinded to the treatment group, under × 200 magnification. The size of inguinal adipocytes was estimated using Win ROOF version 5.02 (MITANI Co, Fukui, Japan).

# Immunohistochemistry

The harvested adipose tissue was stained immunohistochemically using antibody for CD11b (Abcam (dilution, 1:100, Cambridge, UK), as described previously [[2](#_ENREF_2),[7](#_ENREF_7)]. The numbers of CD11b-positive and -negative cells were counted by two independent investigators who were blinded to the treatment group, under × 200 magnification. The proportion of CD11b-positive cells was calculated as the total number of nuclei of CD11b-positive cells divided by the total number of nuclei in a slide. Ten microscopic fields were chosen in three different sections per mouse for examination.

Supplementary Table 1. Sequences of primers used for RT-PCR.

| Gene | Forward (5'-3') | Reverse (5'-3') |
| --- | --- | --- |
| mouse MCP-1 | actgaagccagctctctcttcctc | ttccttcttggggtcagcacagac |
| mouse TNFα | tatggctcagggtccaactc | agcaaaagaggaggcaacaa |
| mouse IL-6 | ccggagaggagacttcacag | ggaaattggggtaggaagga |
| mouse IRS-1 | ccagagtcaagcctcacaca | cccaactcaactccaccact |
| mouse GLUT4 | tccctgttacctccaggttg | ccttgccctgtcaggtatgt |
| mouse CD68 | ttctgctgtggaaatgcaag | agaggggctggtaggttgat |
| mouse F4/80 | ttttcagatccttggccatc | acactggggcacttttgttc |
| mouse adiponectin | tggatctgacgacaccaaaa | cgaatgggtacattgggaac |
| mouse β-actin | tggaatcctgtggcatccatgaaac | taaaacgcagctcagtaacagtccg |
| mouse angiotensinogen | agcatcctcctcgaactcaa | tcttccagagtggcagtcct |

**Reference**

1. Yamamoto K, Shimokawa T, Yi H, Isobe K, Kojima T, et al. (2002) Aging and obesity augment the stress-induced expression of tissue factor gene in the mouse. Blood 100: 4011-4018.

2. Uchida Y, Takeshita K, Yamamoto K, Kikuchi R, Nakayama T, et al. (2012) Stress augments insulin resistance and prothrombotic state: role of visceral adipose-derived monocyte chemoattractant protein-1. Diabetes 61: 1552-1561.

3. Yamamoto K, Takeshita K, Shimokawa T, Yi H, Isobe K, et al. (2002) Plasminogen activator inhibitor-1 is a major stress-regulated gene: implications for stress-induced thrombosis in aged individuals. Proc Natl Acad Sci U S A 99: 890-895.

4. Aoyama T, Takeshita K, Kikuchi R, Yamamoto K, Cheng XW, et al. (2009) gamma-Secretase inhibitor reduces diet-induced atherosclerosis in apolipoprotein E-deficient mice. Biochem Biophys Res Commun 383: 216-221.

5. Cheng XW, Okumura K, Kuzuya M, Jin Z, Nagata K, et al. (2009) Mechanism of diastolic stiffening of the failing myocardium and its prevention by angiotensin receptor and calcium channel blockers. J Cardiovasc Pharmacol 54: 47-56.

6. Takeshita K, Yamamoto K, Ito M, Kondo T, Matsushita T, et al. (2002) Increased expression of plasminogen activator inhibitor-1 with fibrin deposition in a murine model of aging, "Klotho" mouse. Semin Thromb Hemost 28: 545-554.

7. Takeshita K, Satoh M, Ii M, Silver M, Limbourg FP, et al. (2007) Critical role of endothelial Notch1 signaling in postnatal angiogenesis. Circ Res 100: 70-78.
